# Effect of Mobile Apps on Medication Adherence of Type 2 Diabetes Mellitus: A Systematic Review of Recent Studies

**DOI:** 10.7759/cureus.51791

**Published:** 2024-01-07

**Authors:** Abdullah M Hakami, Bader Almutairi, Ahmad S Alanazi, Mohammed A Alzahrani

**Affiliations:** 1 Family Medicine, King Fahad Military Medical Complex, Dammam, SAU; 2 Family Medicine, Imam Abdulrahman Bin Faisal Hospital, Dammam, SAU

**Keywords:** diabetes, mobile app, medication adherence, glycated hemoglobin (hba1c), diabetes type 2

## Abstract

Medication adherence is a critical aspect of managing type 2 diabetes mellitus (T2DM) and achieving optimal clinical outcomes. Mobile app-based interventions have emerged as a promising tool to enhance adherence and glycemic control in T2DM patients. This systematic review aims to evaluate the effectiveness of mobile app interventions in improving medication adherence and glycated hemoglobin among T2DM patients. A comprehensive search was conducted in PubMed, Cochrane Library, and Google Scholar for studies published between September 2018 and September 2023. Studies were included if they were published in English and investigated the effectiveness of mobile apps in enhancing medication adherence among patients with T2DM. Studies were excluded if they included additional interventions, such as electronic pillboxes, phone calls, or SMS text messages, or if they focused on populations with chronic illnesses other than T2DM. Five studies involving 527 participants from diverse geographic locations were included in the review. The findings from the included studies show that mobile-based app interventions can significantly improve medication adherence in patients with T2DM. From the included studies, the mean HbA1c change for the intervention group was -0.664 (95%CI -0.823 to -0.506), while the mean change in HbA1c for the control group was -0.103 (95%CI -0.305 to 0.099). Studies have demonstrated the potential of mobile app-based interventions to enhance medication adherence and improve glycemic control in T2DM; further research is needed to determine the long-term effects of these interventions.

## Introduction and background

Type 2 diabetes mellitus (T2DM) is one of the most common chronic noncommunicable diseases in the ‎world; it significantly affects people's physical, mental, and quality of life. The ‎International Diabetes Federation estimates that the global diabetes prevalence in ‎‎2019 was 9.3% (463 million people), rising to 10.2% (578 million) by 2030 ‎[1].

In the United States, the estimated national cost of diabetes in 2017 was $327 billion, of which ‎‎$237 billion represents direct healthcare expenditures attributed to diabetes, and $90 ‎billion represents lost productivity [2].‎ T2DM is a chronic metabolic condition defined by insulin insensitivity as a result of insulin resistance, declining insulin production, and eventual pancreatic beta-cell failure‎ [3]. Diabetes can lead to a variety of complications that affect several organs and systems in the body. These complications can be classified into two categories: Macrovascular complications (coronary artery disease, peripheral artery disease, and stroke) and microvascular complications (diabetic nephropathy, neuropathy, and retinopathy) [4].‎ To prevent vascular complications in diabetic patients, blood glucose BG levels must ‎be routinely checked and well-regulated (for nonpregnant adults: target fasting BG between 80-130 mg/dL and postprandial less than 180 mg/dL). Nonadherence to medication is common among diabetic patients, which results in ‎uncontrolled diabetes, poor outcomes, worse quality of life, ‎and increased hospitalization risk. Multiple factors contribute to poor medication adherence. Patient factors include education level, income, beliefs, health literacy, mental illness, and cognitive function. External factors involve access to healthcare, complex regimens, and medication side effects. Additionally, miscommunication and poor patient-provider relationships can contribute to nonadherence‎. Medication nonadherence is common among T2DM; studies show that adherence varies depending on the treatment regimen. For example, people taking only oral medications have an adherence rate of 36% to 87%, while those taking both oral medications and insulin or only insulin have an adherence rate of 54% to 81% [5]. In a cross-sectional study, data showed that medication adherence is higher in diabetics alone compared with coexisting hypertension and ‎diabetes; moreover, adherence is higher in educated patients [6].‎ The widespread use of information and communication technologies in the healthcare ‎industry has led to significant advancements in the delivery of healthcare, including ‎the promotion of patient-centered care, raising the standard of care, and educating ‎both patients and healthcare professionals. Information and communication ‎technologies have been utilized to prevent and treat chronic disease and enhance ‎medication adherence. For example, the implementation of telephone ‎coaching in T2DM showed significant improvement in blood glucose reading, HbA1c, and body mass index [7]. Furthermore, data showed that web-based interventions in dietary ‎modification for T2DM reduce hemoglobin A1c and body mass index [8]. Many apps are available to gather health information, provide clinical decision support systems, and aid in medication adherence. Additionally, substantial data support the effectiveness of apps in ‎enhancing lifestyle modification for T2DM patients [9].

Apps have been successful in improving medication adherence in patients with cardiovascular disease, hypertension, asthma, and older adults with ‎coronary heart disease‎ [10-12]. This study thoroughly assesses the effect of a mobile app on medication adherence in patients with T2DM.

## Review

Study objective 

The aim of this study was to conduct a systematic review of clinical studies that ‎employed mobile app-based therapies in T2DM patients to enhance medication ‎adherence.‎

Methodology

Study Design

This systematic review followed PRISMA guidelines to ensure rigorous and transparent reporting of the research process [13]. The review focused on assessing the effectiveness of mobile app-based interventions in improving medication adherence and glycated hemoglobin in individuals with T2DM.

Search Strategy

A systematic and comprehensive search was conducted in major electronic databases, including PubMed, Cochrane Library, and Google Scholar, to identify relevant studies published between September 2018 and September 2023. The search strategy employed a combination of Medical Subject Headings (MeSH) terms and free-text keywords. The search string used the Boolean operators "OR" and "AND" to combine the following key terms: "mobile app," "mobile application," "smartphone application," "medication adherence," "medication compliance," "type 2 diabetes mellitus," and "T2DM."

Inclusion and Exclusion Criteria

This review included studies that met the following criteria: studies published in English between September 2018 and September 2023, studies that investigated the effectiveness of mobile apps in enhancing medication adherence for patients with T2DM, and studies focused on patients or caregivers utilizing a mobile app for T2DM management. Studies were excluded if they reported additional interventions to enhance medication adherence, such as electronic pillboxes, phone calls, or SMS text messages, studies focused on populations with chronic illnesses other than T2DM, and studies did not measure medication adherence.

Data Extraction

For the five studies included in the systematic review, data were extracted using a structured data extraction form. This form included information on the study's characteristics, including the study ID, country, study design, sample size, inclusion criteria, exclusion criteria, and duration of follow-up.

Data synthesis

A qualitative data synthesis approach was employed to analyze and interpret the findings from the included studies. This involved summarizing and comparing the key outcomes, discussing patterns and variations in the results, and examining the implications of the findings. The discussion included a comprehensive analysis of factors contributing to the observed results and their implications for the effectiveness of mobile app-based interventions in enhancing medication adherence and T2DM management.

Results

Search Results

The study selection process comprised two stages: title and abstract screening, followed by full-text assessment. After duplicate removal, the titles and abstracts of 76 identified records were screened independently by two reviewers. Records that did not meet the inclusion criteria were excluded, resulting in the selection of 29 records for full-text assessment. Subsequently, the full texts of these 29 articles were evaluated to determine their eligibility for inclusion in the systematic review. These articles underwent a rigorous full-text assessment. To ensure the robustness of our review process, all articles were assessed by two independent reviewers. Any discrepancies between reviewers were resolved through discussion and consensus among the research team members.

Following this meticulous evaluation process, we ultimately included five studies that met our stringent inclusion criteria in the systematic review [14-18].

**Figure 1:**
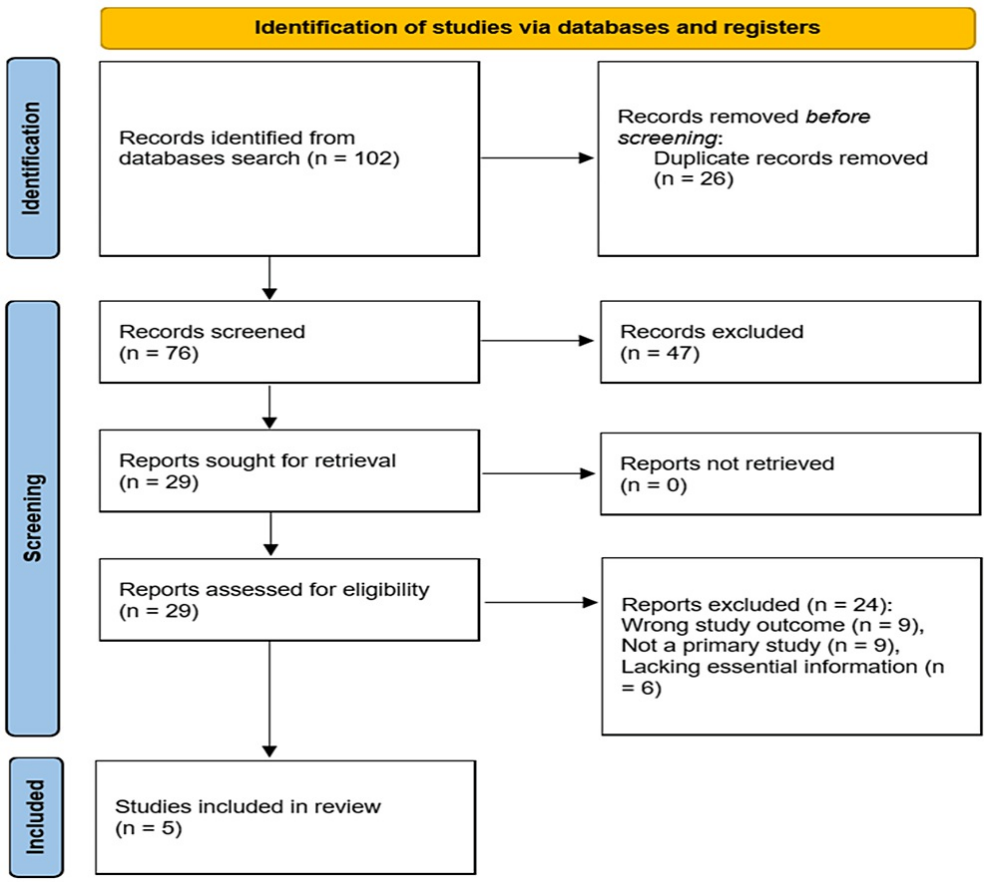
PRISMA flow diagram for summary of the search process

To illustrate the flow of the study selection process, we have adhered to the PRISMA guidelines by providing a detailed flow diagram in this manuscript (Figure 1).

Summary of the included studies

Table 1 provides a concise overview of the five studies, offering critical details about their characteristics. These studies spanned diverse populations and regions, thereby contributing to the robustness of the systematic review.

**Table 1:** Summary of the included studies (n=5)

Study ID	Country	Study design	Sample size	Final sample size	Inclusion criteria	Exclusion	Duration of follow-up
Huang et al. 2019 [14]	Singapore	Randomized control trial	Intervention 25, control 26	Intervention 22, control 19	Type 2 diabetes mellitus (T2DM), on oral or injection hypoglycemic, agent, and English speaker	Pregnant, cognitive impairment, bedridden, and type 1 DM	Three months
Rai et al. 2023 [15]	United States	Pragmatic, quasi-experimental pilot study case-crossover	50 participants	Intervention 17, control 13	Older than 18 with T2DM	Insulin users and patients do not have a smartphone	Three months
Pamungkas et al. 2022 [16]	Indonesia	Randomized control trial	Intervention 30, control 30	Intervention 30, control 30	HbA1c >7%, and age 35-59 y/o	Foot ulcers, chronic renal diseases, and retinopathy	Three months
Poonprapai et al. 2022 [17]	Thailand	Randomized control trial	Intervention 83, control 83	Intervention 78, control 79	≥65 years of age, T2DM, (HbA1c) level>7%, receipt of oral antihyperglycemic agents, and having a family member as a caregiver. The eligible family members were ≥18 years old, a spouse, child, or relative of the patient, and able to participate in this study by using a mobile phone	Patients who received insulin therapy were excluded from the study	Nine months
Yang et al. 2020 [18]	South Korea	Multicenter, cluster-randomized	Intervention 150, control 97	Interventions 145, control 94	≥18 years of age, had T2DM for at least one year, could use mobile phones or internet services at home, and had baseline HbA1c between 7% and 10%	Type 1 diabetes and insulin pump users, subjects with any significant medical disease (e.g., active cancer, recent stroke, or myocardial infarction), subjects with severe diabetic complications (retinopathy, serum creatinine >1.5 for men or >1.4 for women)	Three months

The age range of participants in these studies was fairly wide, varying from 18 to 85 years. The gender distribution also displayed a balance, with no significant differences between intervention and control groups. Various adherence measurement scales, such as the Adherence Starts with Knowledge-12 scale, adherence measured by pill count, and self-efficacy of appropriate medication use scale (SEAMS scores) were utilized to assess medication adherence. The baseline data concerning HbA1c, which is an essential parameter for assessing diabetes management, were measured.

The baseline characteristics of the populations included in these studies are as follows.

Huang [14]: The intervention group had 25 participants, while the control group had 26 participants. Median ages were 51.5 (range: 22-69) in the intervention group and 52 (range: 28-67) in the control group. The gender distribution was 13 females in the intervention group and eight in the control group.

Rai et al. [15]: This crossover study had 50 participants. The median age was 66 (IQR 56.25-73.25).

Pamungkas et al. [16]: The study included 30 participants in both intervention and control groups. The mean age was 56.2 (SD 7.6) in the intervention group and 54.5 (SD 9.2) in the control group.

Poonprapai et al. [17]: This RCT had 80 participants in both the intervention and control groups. The mean age was 67.36 (SD 5.72) in the intervention group and 67.80 (SD 6.18) in the control group.

Yang et al. [18]: This cluster-randomized study had 150 participants in the intervention group and 97 in the control group. The mean age was 54.1 (SD 10.1) in the intervention group and 60.6 (SD 10.2) in the control group.

Reported outcomes of the populations

Table 2 and Table 3 offer a comprehensive analysis of drug adherence and HbA1c levels in both intervention and control groups. The outcomes demonstrate the impact of mobile app-based interventions on medication adherence and glycated hemoglobin.

**Table 2:** Baseline and outcome of medication adherence (n=5)

Study ID	Drug adherence baseline		Drug adherence outcome		Tools for measuring adherence	Result
	Intervention group	Control group	Intervention group	Control group		
Huang 2019 [14]	28.6 SD (5.2)	25.5 SD (4.4)	27.2 SD (5.8)	28.5 SD (7.0)	Adherence starts with Knowledge-12 scale score	P value 0.01
Rai 2023 [15]	No available data	No available data	1.90 95%CI (0.86 to 2.94)	−0.20 95%CI (−0.56 to 0.16)	Self-efficacy of appropriate medication use scale	P value <0.001
Pamungkas 2022 [16]	2.57 SD 1.382	3.03 SD 1.79	4.97 SD 0.999	3.1 SD 607	Medication adherence questionnaire	P value <0.001
Poonprapai 2022 [17]	87.17 SD 2.04	87.28 SD 2.29	91.41 SD 3.57	88.47 SD 2.54	Adherence measured by pill count	P value <0.001
Yang 2020 [18]	4.4 (1.3)	4.7 (1.1)	0.52 (0.31 to 0.74)	0.06 (−0.15 to 0.28)	The six-item Morisky medication adherence scale	P value 0.02

**Table 3:** Baseline and outcome of HbA1c (n=5)

Study ID	A1C baseline		Mean change in A1C from baseline		P value
	Intervention	Control	Intervention	Control	
Huang et al. 2019 [14]	8.7 SD (2.4)	8.6 SD (1.5)	+0.3 SD 1.6	+0.8 SD 2.4	P value 0.57
Rai et al. 2023 [15]	9.05 IQR (8.6%-9.6%)	9.05 IQR (8.6%-9.6%)	−0.69 95% CI (0.36 to 1.02)	−0.35 95%CI (-0.12 to 0.82)	P value 0.30
Pamungkas et al. 2022 [16]	8.043 SD (1.96)	8.553 SD (2.95)	−1.603 SD 1.144	−0.313 SD 2.605	P value <0.001
Poonprapai et al. 2022 [17]	8.68 SD(1.60)	8.7 SD(1.69)	−0.97 SD ± 1.61	−0.12 SD ± 1.66	P value 0.001
Yang et al. 2020 [18]	8 SD (0.8)	7.9 SD (0.8)	−0.63 (−0.77 to −0.50)	−0.28 (−0.42 to −0.13)	P value 0.003

The study by Huang et al. [14] in Singapore revealed a notable change in the Adherence Starts with Knowledge-12 scale scores, with the intervention group displaying improved adherence compared to the control group. The intervention group had a Knowledge-12 scale score mean (SD) of 27.2 (5.8), while the control group had a score of 28.5 (7.0) P value of 0.01. Rai et al.'s study [15] in the United States showed significant improvement in medication adherence with using mobile apps, the mean change of self-efficacy of appropriate medication use scale score was 1.9 for the intervention group and -0.2 for the control group P<0.001

Pamungkas et al.'s study [16] in Indonesia demonstrated significant improvements in medication adherence, as indicated by higher adherence scores in the intervention group. The intervention group had a mean drug adherence score of 4.97 (SD 0.999), while the control group had a score of 3.1 (SD 0.607) with P value < 0.001. Poonprapai et al.'s study [17] in Thailand revealed significant improvement in medication adherence in the intervention group compared with the control group (P value 0.001). Finally, Yang et al. [18] in South Korea showed signification improvement in the six-item Morisky Medication Adherence Scale among the intervention group compared with the control group P value 0.02.

Interventions studied by Pamungkas et al., Poonprapai et al., and Yang et al. [16-18] displayed significant improvement in HbA1c but no significant HbA1c improvement in Huang et al. and Rai et al. studies [14-15]. For the five studies, the mean change in HbA1c for the intervention group was -0.664 95%CI -0.823 to -0.506, while the mean change in HbA1c for the control group was -0.103 95%CI -0.305 to 0.099.

In synthesizing the qualitative data from the included studies, the effectiveness of mobile app-based interventions in improving medication adherence becomes evident. All included studies showed significant improvement in medication adherence with using mobile apps. Moreover, studies showed improvement in glycated hemoglobin with mobile app-based interventions.

Discussion

**Figure 2:**
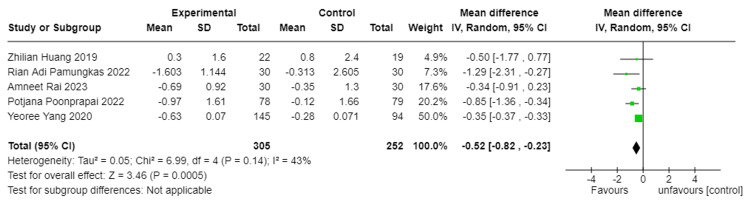
Effect of mobile app-based interventions on HBA1c

The findings of this systematic review offer a comprehensive perspective on the potential of mobile app-based interventions in improving medication adherence and glycated hemoglobin in individuals with T2DM (Figure 2). Our analysis encompassed five studies from different geographical locations.

All five studies showed significant improvement in medication adherence in the intervention groups compared to the control groups. This was measured using different scales in each study, but all indicated increased adherence to the intervention. Improvement in HbA1c levels was mixed. Pamungkas et al., Poonprapai et al., and Yang et al.'s studies [16-18] showed significant improvement in HbA1c, while Huang et al. and Rai et al.'s studies [14,15] showed slight or no improvement. The overall average change in HbA1c for the intervention group was -0.664 compared to -0.103 for the control group.

A variety of medication adherence measurement tools were employed in these five studies. However, the reliability and validity of these tools vary, with some tools being more reliable and valid than others. Rai et al.'s study [15], which utilized different medication adherence tools, revealed that some tools demonstrated significant differences between intervention and control groups, while others did not. For example, the medication possession ratio (MPR) outcome for the intervention group was 112, with an IQR of 100 to 130, while the outcome for the control group was 102, with an IQR of 98.5 to 127 and a P value of 0.37. In contrast, the SEAMS showed significant differences between groups (P value <0.001). These disparate findings underscore the need for standardized adherence measurement tools.

In a systematic review that included 12 studies, the mobile app-based interventions were associated with a clinically significant reduction of HbA1c -0.67 (95% CI -0.30 to -1.03) [19]. Moreover, another systematic review including eight studies showed that mobile app intervention reduced HbA1c by -0.36 (95% Cl - 0.47 to -0.25) compared to non-mobile app users, whereas fasting plasma glucose significantly reduced by 16.75 mg/dL (95% Cl -17.60 to -15.80) [20]. Additionally, smartphone app-based interventions were found to significantly lower HbA1c levels, particularly in those with higher baseline HbA1c levels (≥9%), the mean reduction for the intervention group was -0.91% (95%CI -1.33 to -0.49) [21]. Variable improvement in glycated hemoglobin emphasizes the need for individualized interventions that consider the specific needs of the target population, baseline HbA1c level, and follow-up duration. Heterogeneity in the results of Cheng's systematic review on medication adherence is primarily because of the substantial variation between the adherence measure tools used across the included studies [20]. Mobile apps can deliver various features and functionalities (e.g., medication reminders) and educational materials to help patients take their medications as prescribed and improve lifestyle modification. Mobile apps are seamlessly accessible with features supporting self-monitoring, medication reminders, education, and telehealth, which hold promise for improving medication adherence for various chronic diseases, including T2DM. We need more studies to understand the long-term effect of mobile apps on HBA1C and measure patients' adherence to using the app and their satisfaction with mobile apps.

Overall, our comprehensive analysis of existing research indicates that mobile app interventions hold significant potential for boosting medication adherence and glycated hemoglobin control in individuals with T2DM.

Limitations

The heterogeneity in medication adherence measurement tools used in our included studies limited our ability to draw conclusions through a meta-analysis.

## Conclusions

Our systematic review suggests that mobile app-based interventions hold promising potential for improving medication adherence and enhancing glycemic control in individuals with T2DM. However, more extensive research is warranted to assess the long-term impact of these interventions.

## References

[1] Saeedi P, Petersohn I, Salpea P (2019). Global and regional diabetes prevalence estimates for 2019 and projections for 2030 and 2045: results from the International Diabetes Federation Diabetes Atlas, 9th edition. Diabetes Res Clin Pract.

[2] American Diabetes Association (2018). Economic costs of diabetes in the U.S. in 2017. Diabetes Care.

[3] Olokoba AB, Obateru OA, Olokoba LB (2012). Type 2 diabetes mellitus: a review of current trends. Oman Med J.

[4] Iuga AO, McGuire MJ (2014). Adherence and health care costs. Risk Manag Healthc Policy.

[5] Osborn CY, Egede LE (2012). The relationship between depressive symptoms and medication nonadherence in type 2 diabetes: the role of social support. Gen Hosp Psychiatry.

[6] Jankowska-Polańska B, Świątoniowska-Lonc N, Karniej P, Polański J, Tański W, Grochans E (2021). Influential factors in adherence to the therapeutic regime in patients with type 2 diabetes and hypertension. Diabetes Res Clin Pract.

[7] von Storch K, Graaf E, Wunderlich M, Rietz C, Polidori MC, Woopen C (2019). Telemedicine-assisted self-management program for type 2 diabetes patients. Diabetes Technol Ther.

[8] Dening J, Islam SM, George E, Maddison R (2020). Web-based interventions for dietary behavior in adults with type 2 diabetes: systematic review of randomized controlled trials. J Med Internet Res.

[9] Larbi D, Randine P, Årsand E, Antypas K, Bradway M, Gabarron E (2020). Methods and evaluation criteria for apps and digital interventions for diabetes self-management: systematic review. J Med Internet Res.

[10] Xu H, Long H (2020). The effect of smartphone app-based interventions for patients with hypertension: systematic review and meta-analysis. JMIR Mhealth Uhealth.

[11] Ramsey RR, Plevinsky JM, Kollin SR, Gibler RC, Guilbert TW, Hommel KA (2020). Systematic review of digital interventions for pediatric asthma management. J Allergy Clin Immunol Pract.

[12] Park LG, Ng F, K Shim J, Elnaggar A, Villero O (2020). Perceptions and experiences of using mobile technology for medication adherence among older adults with coronary heart disease: a qualitative study. Digit Health.

[13] Page MJ, McKenzie JE, Bossuyt PM (2021). The PRISMA 2020 statement: an updated guideline for reporting systematic reviews. BMJ.

[14] Huang Z, Tan E, Lum E, Sloot P, Boehm BO, Car J (2019). A smartphone app to improve medication adherence in patients with type 2 diabetes in Asia: feasibility randomized controlled trial. JMIR Mhealth Uhealth.

[15] Rai A, Riddle M, Mishra R, Nguyen N, Valine K, Fenney M (2023). Use of a smartphone-based medication adherence platform to improve outcomes in uncontrolled type 2 diabetes among veterans: prospective case-crossover study. JMIR Diabetes.

[16] Pamungkas RA, Usman AM, Chamroonsawasdi K, Abdurrasyid Abdurrasyid (2022). A smartphone application of diabetes coaching intervention to prevent the onset of complications and to improve diabetes self-management: a randomized control trial. Diabetes Metab Syndr.

[17] Poonprapai P, Lerkiatbundit S, Saengcharoen W (2022). Family support-based intervention using a mobile application provided by pharmacists for older adults with diabetes to improve glycaemic control: a randomised controlled trial. Int J Clin Pharm.

[18] Yang Y, Lee EY, Kim HS, Lee SH, Yoon KH, Cho JH (2020). Effect of a mobile phone-based glucose-monitoring and feedback system for type 2 diabetes management in multiple primary care clinic settings: cluster randomized controlled trial. JMIR Mhealth Uhealth.

[19] Wu Y, Yao X, Vespasiani G (2017). Mobile app-based interventions to support diabetes self-management: a systematic review of randomized controlled trials to identify functions associated with glycemic efficacy. JMIR Mhealth Uhealth.

[20] Chong CJ, Bakry MM, Hatah E, Mohd Tahir NA, Mustafa N (2023). Effects of mobile apps intervention on medication adherence and type 2 diabetes mellitus control: a systematic review and meta-analysis. J Telemed Telecare.

[21] He Q, Zhao X, Wang Y, Xie Q, Cheng L (2022). Effectiveness of smartphone application-based self-management interventions in patients with type 2 diabetes: a systematic review and meta-analysis of randomized controlled trials. J Adv Nurs.

